# Coupling a DNA-Based Machine with Glucometer Readouts for Amplified Detection of Telomerase Activity in Cancer Cells

**DOI:** 10.1038/srep23504

**Published:** 2016-03-24

**Authors:** Wenjing Wang, Shan Huang, Jingjing Li, Kai Rui, Jian-Rong Zhang, Jun-Jie Zhu

**Affiliations:** 1 State Key Laboratory of Analytical Chemistry for Life Science and Collaborative Innovation Center of Chemistry for Life Sciences, School of Chemistry and Chemical Engineering, Nanjing University, Nanjing 210093, China; 2 School of Chemistry and Life Science, Nanjing University Jinling College, Nanjing 210089, China; 3 Department of Radiology, Affiliated Hospital of Xuzhou Medical College, Xuzhou 221006, China; 4 School of Pharmacy, Ningxia Medical University, Yinchuan 750004, China

## Abstract

The strong correlation between cancer and telomerase activity has inspired the development of new strategies to evaluate telomerase activity. Here, a personal glucose meter (PGM) system that uses DNA-based machine amplification to detect telomerase in cancer cells is reported. In this assay, telomerase elongation products are amplified in the form of another type of product by a DNA-based machine. This process can only be activated by the hybridization of the extended telomerase substrate (TS) probe and the complementary primer in the presence of telomerase. The obtained products are then transformed to glucose-related signals via a three-component assay, which enables the simple use of a PGM to indirectly quantify the telomerase activity. The proposed method realizes sensitive telomerase activity detection down to 20 HeLa cells with a significantly enhanced dynamic range. Additionally, short telomerase elongation products, such as telomerase substrate probes with two repetitive sequences, that cannot be detected using the most widely used telomeric repeat amplification protocol assay were detected.

Telomerase plays a vital role in cancer and ageing^1^,^2^,^3^. Telomerase is a reverse transcriptase composed of a protein and a template RNA component. Telomerase uses an internal RNA template to maintain the length of telomeres by adding repetitive sequences to the end of linear chromosomes^4^,^5^,^6^,^7^. In normal cells, the length of telomeres gradually decreases during the process of cell division due to the end replication problem^8^. When a critical length is reached, a DNA damage signal is activated, and cellular senescence is induced^9^,^10^. However, in more than 85% of human cancer types, this telomere length-induced cellular senescence is prevented due to the over-expression of telomerase, enabling the infinite proliferation of these cells. Consequently, telomerase is a promising biomarker and a potential therapeutic target. The detection of telomerase activity is not only valuable for clinical tumour diagnosis and therapy but is also significant for developing telomerase-targeted drugs^11^,^12^,^13^.

Extensive research efforts are focused on evaluating telomerase activity. Among the strategies used to detect telomerase activity, polymerase chain reaction (PCR)-based telomeric repeat amplification protocol (TRAP) is the most frequently used^3^,^14^,^15^,^16^. Although this method is extraordinary sensitive for the detection of telomerase activity at the single-cell level, quantification is difficult, and precise temperature cycling control is required. Additionally, this method is not applicable to the detection of short telomerase elongation products, such as telomerase substrate primers with two repetitive sequences of (TTAGGG)^17^. Alternative methods seeking to resolve these challenges include *in situ* monitoring methods that use gold nanoparticles and mesoporous silica nanoparticles^18^,^19^, colorimetric^20^,^21^,^22^, fluorescent^23^,^24^,^25^,^26^,^27^,^28^, electrochemistry-based methods^29^,^30^,^31^,^32^, electrochemiluminescence-based methods^33^,^34^, surface plasmon resonance (SPR) methods^35^,^36^, and enzyme-linked immunosorbent (ELISA) assays^37^. These methods are useful platforms for telomerase activity detection and related research, but the use of relatively complex instruments limits their further application in remote and less-developed areas where medical facilities and resources are limited.

Personal glucose meters (PGMs) are promising signal output candidates for detection that are widely accessible. Due to their pocket size, low cost, simple operation and rapid response, PGMs have been successfully used for the portable and sensitive detection of various targets ranging from small molecules, DNA, proteins^38^,^39^,^40^,^41^. Encouraged by this, two assays using PGM to evaluate telomerase activity have been reported recently, employing screen-printed gold electrodes (SPGE)^42^, or mesoporous silica nanoparticles (MSN)^43^. Although they expand the application of PGM in telomerase activity detection undoubtedly, some shortcomings still exist for clinical use. For example, the possibilities of false negative results will increase by using these methods since the telomerization took place on the surface of the SPGE or MSN, which is easily contaminated by RNase to inhibit the telomerase activity. Thus, we would expect the telomerization reaction to take place in a close tube while not on the surface or dose not involve the nanoparticle. On the other hand, because the intrinsic dynamic range of PGMs for glucose is 0.6–33 mM, it is difficult to exploit PGMs to directly detect targets at low concentration levels. Thus, signal amplification is usually needed. Due to the specific interaction of complementary nucleotides, DNA has been used as the building block to perform machine-like isothermal amplification. Similar to polymerase chain reaction (PCR) amplification, DNA-based machine amplification requires a substrate strand as the “track”. In the presence of a partially complementary primer (i.e., fuel strand) and polymerase, the primer can be extended along with the substrate. A specifically designed nicking site on the duplex is recognized and cleaved by an endonuclease to form a nick, which initiates the next polymerization cycle. The DNA strand displaced from the track is the “product” of the machine. The complete replication, scission and displacement machine-like process results in a large amount of “product”. DNA-based machines are powerful tool for signal amplification and have attracted increasing interest in recent years^44^,^45^.

Here, we exploited a widely commercialized PGM to develop a novel method for the detection of telomerase activity in cell extracts based on a DNA-based machine that further improves the dynamic range and sensitivity. In this strategy, a tailored TS probe is the “track” of the machine and is first extended by telomerase. Hybridization between the extended sequence and the complementary probe results in subsequent operation of the machine via strand replication, scission, and displacement. Consequently, a large number of “telomerase-related products” are obtained and can be quantified in a three-component sandwich assay format that involves a biotin-modified capture probe DNA assembled in a 96-well plate and a reporter DNA probe conjugated with invertase. The two probes hybridize with the product at its two ends. Conversion of sucrose to glucose by invertase enables signal readout by the PGM. The execution of this procedure only involves mixing, incubation and, finally, collection of the signals using the PGM. The low cost and simple operation of this method will expand access to telomerase detection, particularly in remote areas where medical facilities and resources are limited. This method also possesses the potential to be developed into a point of care (POC) diagnostic device for telomerase detection.

## Results and Discussion

**Figure 1:**
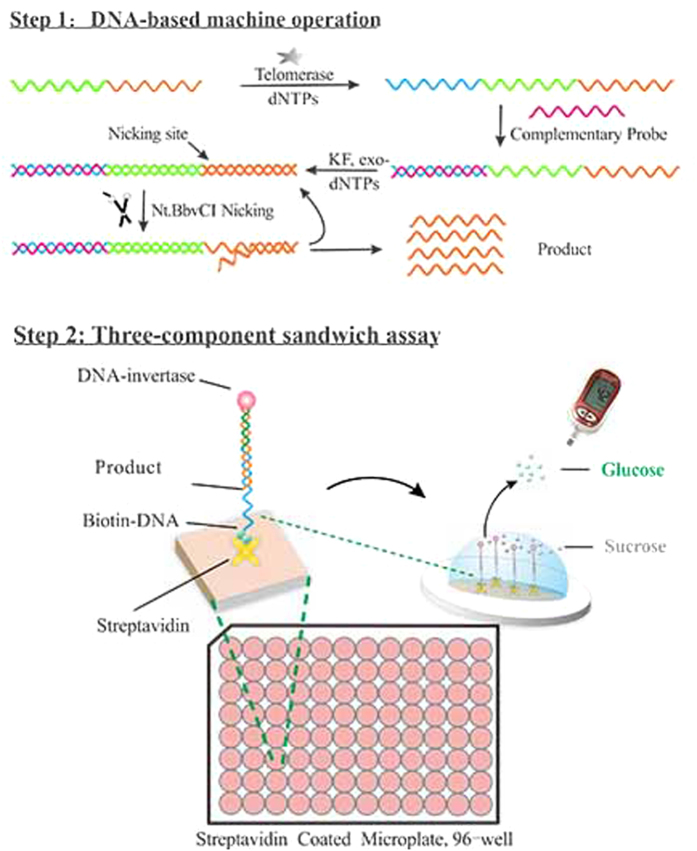
Principle of telomerase activity detection using DNA-based machine amplification and PGM readout.

In this method, by mixing the sample with enzymes, telomerase elongation products are first amplified in the form of another type of product with amplification of a DNA-based machine in a single tube (Step 1 in Fig. 1). The obtained products are then transformed to glucose-related signals via a three-component assay in 96-well plate, which enables the simple use of a PGM to indirectly quantify the telomerase activity (Step 2 in Fig. 1). For detailed explanation, please see Supporting Information.

### Feasibility of the Proposed Method

**Figure 2:**
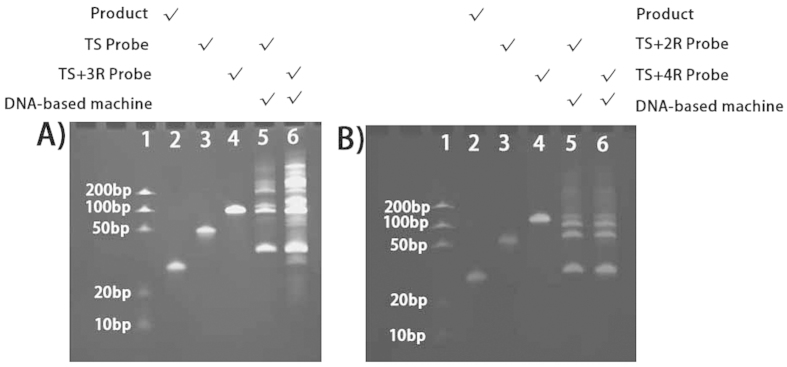
Analysis of assay products by ethidium bromide-stained 12% denaturing polyacrylamide gel electrophoresis (PAGE). (**A**) Lane 1: DNA ladder; Lane 2: 2 *μ*M synthetic products of the DNA-based machine; Lane 3: 1 *μ*M TS probe; Lane 4: 1 *μ*M TS + 3R probe; Lane 5: products of the DNA-based machine triggered by the 25 nM TS probe; Lane 6: products of the DNA-based machine triggered by the 25 nM TS + 3R probe; (**B**) Lane 1: DNA ladder; Lane 2: 2 *μ*M synthetic products of the DNA-based machine; Lane 3: 1 *μ*M TS + 2R probe; Lane 4: 1 *μ*M TS + 4R probe; Lane 5: products of the DNA-based machine triggered by the 25 nM TS + 2R probe; Lane 6: products of the DNA-based machine triggered by the 25 nM TS + 4R probe.

Initially, we employed the synthetic telomerase elongation products TS + 2R, TS + 3R, and TS + 4R (mimetic telomerase extension products), which correspond to the TS probe extended by two, three and four telomeric repetitive sequences of (TTA GGG) to evaluate the feasibility of the proposed method. The feasibility of the first step was verified by denaturing gel electrophoresis. In Fig. 2A, the synthetic products of the DNA-based machine were run in Lane 2 as a standard. Compared with TS + 3R probe only (Lane 4), a band at the bottom of Lane 6 corresponding to the oligonucleotide of Lane 2 was clearly produced by the DNA-based machine when triggered by the TS + 3R probe but not by the TS probe (Lane 5). This result suggests that the TS + 3R probe hybridized with the complementary probe, and the remaining part of TS + 3R probe was replicated by the polymerase and cleaved by the exonuclease to release the products. Thus, the proposed DNA-based machine was successfully operated using the synthetic telomerase elongation product of the TS + 3R probe. To further verify the feasibility of the method, other TS probes with different telomeric repeats were introduced. As shown in Fig. 2B, the products of the TS + 2R probe-triggered DNA-based machine (Lane 5) and the TS + 4R probe-triggered DNA-based machine (Lane 6) resulted in clear bands at the same positions as the synthetic products of the DNA-based machine (Lane 2), confirming the feasibility of our design for both TS + 2R probes and other synthetic telomerase elongation products. In contrast to the TRAP assay, this method can be used to detect short telomerization products (TS + 2R).

**Figure 3:**
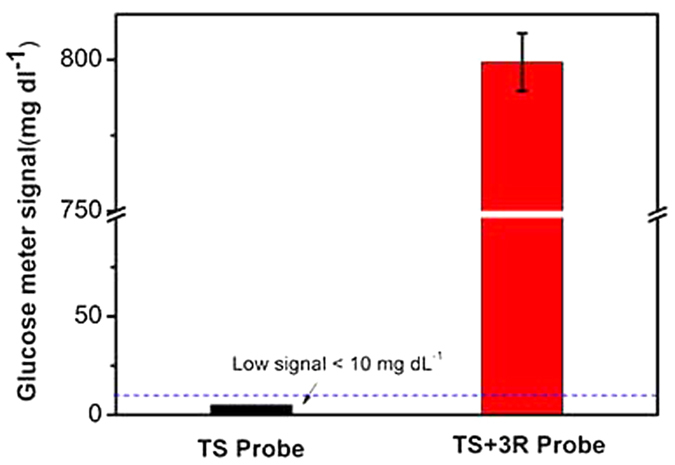
Feasibility analysis of the PGM detection of the DNA-based machine products triggered by 50 nM probe. The signal of nearly 800 mg/dL was calculated based on a test of the PGM with a 2-fold dilution of the sample. The error bars represent the standard deviation of three repeated measurements. The signal below the dash line represents less than 10 mg/dL and shows as “LO” in the PGM.

After confirming the successful operation of the DNA-based machine, we further evaluated the feasibility of the second step using PGM. The DNA-based machine products triggered by 50 nM TS + 3R probe (i.e., a mimetic telomerase extension product) and reporter DNA were sequentially added to the capture DNA modified 96-well plate. The PGM signal reached 800 mg/dL (Fig. 3) due to the high amplification efficiency of the DNA-based machine and the efficiency of the subsequent three-component sandwich assay in the 96-well plate. By contrast, the control sample corresponding to the 50 nM TS probe-triggered DNA-based machine product produced a non-detectable signal because the unextended telomerase substrate strand failed to operate the DNA-based machine to produce the product used as a linker in this step. As clearly illustrated in Fig. 3, the PGM signal of the control sample was below the detection limit. Together with the results of the denaturing gel electrophoresis analysis, the PGM signals confirmed the feasibility of this method for sensing synthetic telomerase elongation products.

**Figure 4:**
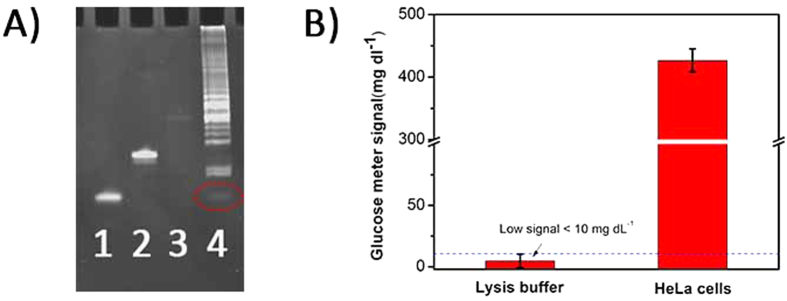
Feasibility analysis for sensing telomerase in cell extracts. (**A**) Image of ethidium bromide-stained 12% denaturing polyacrylamide gel electrophoresis (PAGE). Lane 1: 2 *μ*M synthetic products of the DNA-based machine; Lane 2: 1 *μ*M TS probe; Lane 3: DNA-based machine products triggered by lysis buffer; Lane 4: DNA-based machine products triggered by the telomerase elongation products of 1000 cells; (**B**) PGM signals of the different samples. The error bars represent the standard deviation of three repeated measurements. The signal below the dash line represents less than 10 mg/dL and shows as “LO” in the PGM.

Next, we employed telomerase in HeLa cell extracts to verify the capability for sensing telomerase in cell extracts. The TS probe was initially extended by telomerase for 1 h. As expected, a band ascribed to the production of the DNA-based machine triggered by the telomerase elongation products of 1,000 HeLa cells (Lane 4, Fig. 4A) was observed at the same position as the synthetic products of the DNA-based machine (Lane 1, Fig. 4A). This observation indicated that the TS probe was extended by telomerase and activated the DNA-based machine. However, in the absence of telomerase, the band could not be observed (Lane 3, Fig. 4A). Identical results were obtained for the PGM step, as illustrated in Fig. 4B. Compared to the weak signal for the lysis buffer sample, the signal for the 1,000-cell extract was significantly enhanced. Taken together, these results clearly demonstrated the feasibility of telomerase activity sensing.

### Optimization of the Capture Probe Concentration on the Plate

**Figure 5:**
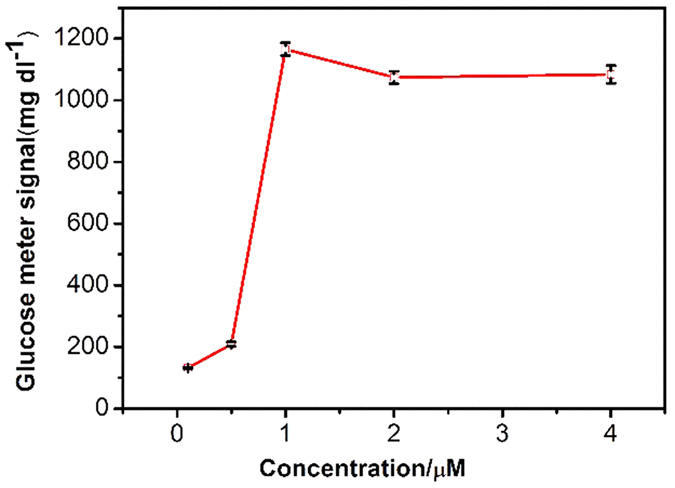
Optimization of the capture probe concentration assembled on the streptavidin-coated 96-well plate. The signal of greater than 600 mg/dL was calculated from a test of the PGM at several fold dilutions of the sample. The error bars represent the standard deviation of three repeated measurements.

Because the density of the capture probe on the plate significantly influences performance, the capture probe concentration was optimized to obtain the best signal using 1 *μ*M synthetic telomerase elongation product (TS + 3R probe). As shown in Fig. 5, the PGM signal increased greatly with the increase in the capture probe concentration from 0 to 1 *μ*M, as the higher probe concentration on the plate led to greater hybridization of DNA-invertase conjugates. However, with further increases in the probe concentration, the signal remained relatively constant because the plate was saturated by the capture probe. Therefore, the optimized concentration of capture probe for plate modification of 1 *μ*M was used in subsequent experiments.

### Synthetic Telomerase Elongation Product Detection

**Figure 6:**
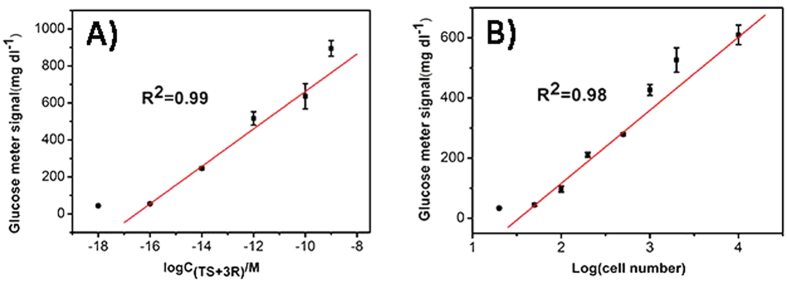
(**A**) PGM signals corresponding to the analysis of the TS + 3R probe at different concentrations: 1 aM, 0.1 fM, 10 fM, 1 pM, 0.1 nM, and 1 nM. The signal of greater than 600 mg/dL was calculated from a test of the PGM with several fold dilutions of the sample. (**B**) The PGM signals corresponding to the analysis of extracts of different numbers of HeLa cells: 20, 50, 100, 200, 500, 1000, 2000, and 10000 cells. The error bars represent the standard deviation of three repeated measurements.

Before evaluating the telomerase activities of the cell extracts, we roughly assessed the sensitivity of the method based on the synthetic telomerase elongation product (TS + 3R probe). As shown in Fig. 6A, the PGM signal was highly dependent on the concentration of the TS + 3R probe. The PGM signal increased with increasing TS + 3R probe concentration because higher concentrations of the TS + 3R probe produced more products via the DNA-based machine for capture on the plate. The PGM intensity exhibited a linear correlation with the logarithm of the TS + 3R probe concentration over a large dynamic range of 7 orders of magnitude, from 0.1 fM to 1 nM. The directly measured detection limit of 0.1 fM was readily achieved without any background subtraction. This result suggests that this strategy is efficient for the sensitive detection of synthetic telomerase elongation product and has potential for sensing telomerase activity in cell extracts.

### Telomerase Activity Detection in Cell Extracts

HeLa cell extracts were used to assess the sensitivity of the proposed method. Because the concentration of the DNA-based machine product depends on the telomerase activities of the cell extracts, the PGM signal should be closely related to the number of cancer cells. As expected, the PGM signal increased with increasing cell number from 20 to 10,000 (Fig. 6B), clearly demonstrating that higher telomerase activity produces more DNA-based machine products, and, consequently, higher PGM signals. A linear relationship between the PGM signal and the logarithm of the number of HeLa cells was observed in the range of 50–10,000 cells. A readily detectable PGM signal was distinguishable from the background signal (S/N = 3) in the presence of telomerase-containing extracts of 20 HeLa cells. This sensitivity is comparable to those of most elaborate instrument-based methods^25^,^34^,^46^. Simultaneously, the proposed method also exhibits a significantly enhanced dynamic range compared to instrument-based methods.

**Figure 7:**
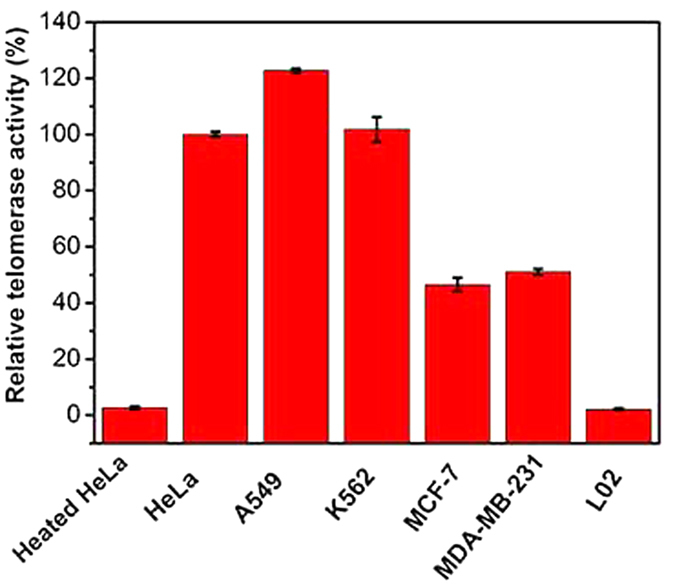
Evaluation of the telomerase activity of the different cell lines using the proposed method. The error bars represent the standard deviation of three repeated measurements.

To ensure practicability for clinical diagnoses, we evaluated the telomerase activities of five different cancer cell lines and one normal cell line, including the human cervical cancer cell line HeLa, the lung cancer cell line A549, the leukaemia cell line K562, the breast cancer cell lines MCF-7 and MDA-MB-231, and normal liver cell line L02. As shown in Fig. 7, high PGM signals were obtained for all of these cell lines, consistent with the over-expressed telomerase activity in the human cancer cell line^3^. By contrast, the heated HeLa cell sample and normal liver cell line exhibited only a weak background signal due to the loss and inexistence of telomerase activity. Similar to previous report^21^, the telomerase activities of the HeLa and A549 cells were higher than those of the MCF-7, K562 and MDA-MB-231 cells, indicating the reliability of our method in evaluating the telomerase activities of different cell lines.

## Conclusions

In conclusion, we have proposed a novel strategy for detecting telomerase activity in cell extracts via DNA-based machine amplification and PGM readouts. The practicality of the proposed method was verified by measuring the telomerase activities of 5 different cell lines (HeLa, A549, K562, MCF-7, and MDA-MB-231). By exploiting the high efficiency of DNA-based machine amplification and the enzymatic turnover of invertase, a sensitive method with a detection limit as low as 20 HeLa cells and an enhanced dynamic range of 50 to 10,000 cells was developed. Additionally, this method was able to detect short telomerization products that cannot be detected by the TRAP assay. More importantly, the execution of this procedure only involves mixing, incubation and, finally, collection of the signals using the PGM. The low cost and simple operation of this method will expand access to telomerase detection, particularly in remote areas where medical facilities and resources are limited. This method also possesses the potential to be developed into a point of care (POC) diagnostic device for telomerase detection. What we have to point out at last is that, careful attention should be paid to the false positive results due to the contamination by normal cells with telomerase activity when considering this method for relavant clinial use, for example lymphocytes in patients with inflammatory diseases^47^. As long as the users are aware of the potential positive results, other microscopic cytopathology methods should be combined to get a more reliable result under this circumstance.

## Methods

### Materials

NEB buffer 4 and Nt.BbvCI endonuclease were obtained from New England BioLabs (Ipswich, MA, USA), and the polymerase Klenow fragment exo- was purchased from Thermo Fisher Scientific Inc. (Waltham, MA, USA). Streptavidin-coated 96-well plates were purchased from BEAVER Nano-Technologies (Suzhou, China). 3-[(3-Cholamidopropyl)dimethylammonio]-1-propanesulfonic acid (CHAPS), tris(2-carboxyethyl)phosphine hydrochloride (TCEP), Tween 20, and invertase from baker’s yeast were purchased from Sigma-Aldrich (St. Louis, MO, USA). Ethylene glycol, bis(aminoethyl ether)-N, N, N’, N’ tetraacetic acid (EGTA), glycerol, sulfosuccinimidyl-4-(N-maleimidomethyl)-cyclohexane-1-carboxylate (sulfo-SMCC), bovine serum albumin (BSA), deoxynucleotide triphosphate (dNTP) solution mixture, 40% acrylamide mix solution, ammonium persulfate (APS), and 1,2-bis(dimethylamino)-ethane (TEMED) were obtained from Sangon Biotechnology Co. Ltd. (Shanghai, China). All other chemicals were of analytical grade and were used as received without purification. All water used in this work was RNase-free.

The oligonucleotides used in this assay (Table 1) were synthesized and purified by Sangon Biotechnology Co. Ltd. (Shanghai, China). Buffer I (0.1 M NaCl, 0.1 M sodium phosphate buffer, pH 7.3, 0.05% Tween-20) was used throughout the experiment.

### DNA-Invertase Conjugate Preparation

The conjugates of thiol-modified reporter DNA and invertase were prepared according to the literature with some modifications^38^. In a typical assay, 30 *μ*L of 0.1 mM thiol-modified reporter DNA solution was mixed with 2 *μ*L of 1 M sodium phosphate buffer (pH 5.5) and 2 *μ*L of 3 mM TCEP solution at room temperature to activate the thiol-modified DNA strands. After reaction for 1 h, the excess TCEP was removed in a 10-K ultrafiltration tube 8 times using Buffer I without Tween-20. Simultaneously, to 1 mg of sulfo-SMCC, 400 *μ*L of 2 mg/mL invertase in Buffer I without Tween-20 solution was added and mixed well. The mixture was then plate on a the shaker for 1 h. The mixture was subsequently purified in a 100-K ultrafiltration tube 8 times using Buffer I without Tween-20. The above two purified solutions were mixed and maintained at room temperature for 48 h. To remove the excess thiol-modified DNA, the solution was purified in a 100-K ultrafiltration tube 8 times in Buffer I without Tween-20 and stored at 4 °C for further use.

### Cell Culture and Telomerase Extraction

All cell lines were obtained from KeyGEN BioTECH (Nanjing, China). HeLa cells, A549 cells, MCF-7 cells, MDA-MB-231 cells and L02 cells were cultured in DMEM medium (Gibco, Grand Island, NY) containing 10% fetal calf serum (Gibco, Grand Island, NY), penicillin (100 *μ*g/mL), and streptomycin (100 *μ*g/mL) at 37 °C in a 5% CO_2_ atmosphere. K562 cells were cultured in RPMI-1640 medium (Gibco, Grand Island, NY) containing 10% fetal calf serum (Gibco, Grand Island, NY), penicillin (100 *μ*g/mL), and streptomycin (100 *μ*g/mL) at 37 °C in a 5% CO_2_ atmosphere. The cells were collected in the exponential phase of growth, and 1 million cells were dispersed in a 1.5-mL EP tube, washed twice with ice-cold phosphate-buffered saline (pH 7.4) solution and resuspended in 200 *μ*L of ice-cold CHAPS lysis buffer (10 mM Tris-HCl, pH 7.5, 1 mM MgCl_2_, 1 mM EGTA, 0.5% (W/V) CHAPS, 10% (V/V) glycerol). The lysates were incubated on ice for 30 min and then centrifuged at 12,000 rpm at 4 °C for 20 min. The upper cleared lysate solution was carefully transferred to a fresh RNase-free tube, flash frozen and stored at −80 °C before use.

### DNA-Based Machine Operation

1. Telomerase extension reaction. Twenty microlitres of telomerase extract was added to 30 *μ*L of extension buffer containing TRAP buffer (20 mM Tris-HCl, pH 8.3, 1.5 mM MgCl_2_, 63 mM KCl, 0.005% (V/V) Tween 20, 1 mM EGTA), 1 mM dNTPs and 20 nM TS probe. The mixture was incubated at 30 °C for 1 h, followed by 15 min at 95 °C to inactivate the telomerase. For the negative control experiments, the telomerase extracts were first heated at 95 °C for 15 min.
2. DNA-based machine operation trigged by the telomerization product. One microlitre of the telomerization product solution containing different numbers of cells or 1 *μ*L of synthetic telomerase elongation products of different concentrations was mixed with 44 *μ*L of buffer solution containing NEB buffer 4 (50 mM KAc, 20 mM Tris-acetate, 10 mM Mg(Ac)_2_, 1 mM DTT, pH 7.9), 20 nM complementary DNA probe. After incubation for 1 h to allow the telomerization product or the synthetic telomerase elongation product to sufficiently hybridize with the complementary probe. 2 *μ*L of Nt.BbvCI endonuclease (20 U), 2 *μ*L of polymerase Klenow fragment exo- (10 U) and 1 *μ*L of 25 mM dNTP solution were added to the mixture. The mixture was incubated at 37 °C for 1 h to operate the DNA-based machine, and the machine was then stopped by incubating the mixture at 80 °C for 20 min.

### Sensor Fabrication and Telomerase Detection Using PGM

All incubations were performed in a shaker at 37 °C. Fifty microlitres of different concentrations of capture probe in Buffer I were added to a streptavidin-coated 96-well plate and incubated for 30 min. The plate was washed with Buffer I 3 times, and 50 *μ*L of 2 mg/mL BSA solution was then added to block nonspecific binding sites and remove unbound capture probe. After reaction for 1 h, the plate was washed 3 times with Buffer I. To the above-mentioned wells, 50 *μ*L of the DNA-based machine product was added and incubated for 1 h, and the plate was further washed with Buffer I 3 times. Then, 50 *μ*L of 5 mg/mL reporter DNA-invertase conjugate in Buffer I was added and incubated for another 1 h. After washing the wells 3 times with Buffer I, 50 *μ*L of 0.5 M sucrose in Buffer I was added to the wells and incubated for 16 h. A 5-*μ*L aliquot of the final solution was measured using the PGM.

### Gel Electrophoresis

DNA-based machine operation and the feasibility of the proposed method were verified by electrophoresis on a 12% denaturing polyacrylamide gel. Five microlitres of DNA-based machine product were loaded on the gel. Electrophoresis was performed in TBE buffer at 100 V for 70 min at 45 °C. After separation, the gel was stained with ethidium bromide and imaged with a fluorescence gel imaging system.

## Additional Information

**How to cite this article**: Wang, W. *et al*. Coupling a DNA-Based Machine with Glucometer Readouts for Amplified Detection of Telomerase Activity in Cancer Cells. *Sci. Rep*. **6**, 23504; doi: 10.1038/srep23504 (2016).

## Supplementary Material

Supplementary Information

## Figures and Tables

**Table 1:** Probes and sequences of the oligonucleotides used in this study.

Name	Sequence (5′-3′)
TS probe	AATAATCCCAACCCGCCCTACCCGCTGAGGTTAATCCGTCGAGCAGAGTT
TS + 2R probe	AATAATCCCAACCCGCCCTACCCGCTGAGGTTAATCCGTCGAGCAGAGTTagggttagggtt
TS + 3R probe	AATAATCCCAACCCGCCCTACCCGCTGAGGTTAATCCGTCGAGCAGAGTTagggttagggttagggtt
TS + 4R probe	AATAATCCCAACCCGCCCTACCCGCTGAGGTTAATCCGTCGAGCAGAGTTagggttagggttagggttagggtt
Capture probe	Biotin-AAT AAT CCC AAC CC
Reporter probe	GCCC TAC CCG CTGA-(CH_2_)_6_-SH
Complementary probe	aaccctaaccctaaccct
