# Supplementary material for: Coupling a DNA-Based Machine with Glucometer Readouts for Amplified Detection of Telomerase Activity in Cancer Cells
Source: Sci Rep. 2016 Mar 24;6:23504. doi: 10.1038/srep23504 (PMC4806334; doi:10.1038/srep23504)
Supplement: Supplementary Information [file srep23504-s1.pdf]

Supporting Information

Coupling a DNA-Based Machine with Glucometer Readouts for Amplified Detection of Telomerase Activity in Cancer Cells

Wenjing Wang[1], Shan Huang[1,4], Jingjing Li[3], Kai Rui[1], Jian-Rong Zhang[1,2,*], and Jun-Jie Zhu[1,*]

[1] State Key Laboratory of Analytical Chemistry for Life Science and Collaborative Innovation Center of Chemistry for Life Sciences, School of Chemistry and Chemical Engineering, Nanjing University, Nanjing 210093, China.

[2] School of Chemistry and Life Science, Nanjing University Jinling College, Nanjing 210089, China.

[3] Department of Radiology, Affiliated Hospital of Xuzhou Medical College, Xuzhou 221006, China.

[4] School of Pharmacy, Ningxia Medical University, Yinchuan 750004, China.

**Corresponding authors: jrzhang@nju.edu.cn (J. R. Z.) and jjzhu@nju.edu.cn (J. J. Z.).*

Detailed explanation of the principle:

The principle of telomerase activity detection using DNA-based machine signal amplification and PGM readout is illustrated in Figure 1. The entire process includes two parts: (i) the automatic operation of the DNA-based machine triggered by telomerization products or synthetic telomerase elongation products; and (ii) sensor fabrication and telomerase activity detection using the PGM in a 96-well plate. In the first part, telomerase triggers the DNA-based machine because hybridization of the substrate with the complementary primer depends on extension of the substrate by telomerase. A tailored TS probe, the components of which are illustrated in green and orange in Figure 1, was employed as the "track" of the machine. The green part is the telomerase substrate primer obtained from the literature[1], and the orange part is the complementary strand, of which was designed to contain a nicking site recognized by Nt.BbvCI endonuclease. Endonuclease Nt.BbvCI could recognize the specific DNA sequence 5'…CC/TCAGC…3' at the slash position, a lot of amplification methods have been established based on this mechanism.[2, 3] In the presence of the telomerase and dNTP mixture, the TS probe is extended by the addition of repetitive sequences of TTAGGG to the 3' end of the probe. After the extension reaction, the complementary probe composed of three (tcccaa) repetitive sequences is added to the mixture. Upon the formation of the duplex strand, the polymerase Klenow fragment exo- utilizes the dNTPs to replicate the TS probe in the reverse direction. Next, the nicking site in the replication strand of the orange part is recognized and cleaved by the Nt.BbvCI exonuclease, producing a nick in the duplex strand and initiating a secondary polymerization cycle while displacing the sequence. Consequently, a large amount of product is produced by the DNA-based machine. By simply mixing the telomerase samples with the buffer and enzyme solution in a single tube, the amplified telomerase elongation product is obtained for the subsequent three-component sandwich assay. More importantly, the DNA-based machine

can also be successfully operated with the TS+2R probe; thus, even extension of the telomerase substrate probe by two repetitive sequences can activate the machine, and the short telomerase elongation product can be detected. In the second part of the telomerase detection process, a biotin-modified capture probe is added to a streptavidin-coated 96-well plate. The DNA-based machine products hybridize with the capture probe and the reporter DNA probe-invertase conjugates. The conversion of sucrose to glucose by invertase is detected by the PGM and ultimately indirectly reflects telomerase activity.

References:

(1) Kim, N. W.; Piatyszek, M. A.; Prowse, K. R.; Harley, C. B.; West, M. D.; Ho, P. L. C.; Coviello, G. M.; Wright, W. E.; Weinrich, S. L.; Shay, J. W. *Science* **1994**, *266*, 2011.

(2) Shlyahovsky, B.; Li, D.; Weizmann, Y.; Nowarski, R.; Kotler, M.; Willner, I. *J. Am. Chem. Soc.* **2007**, *129*, 3814.

(3) Li, D.; Wieckowska, A.; Willner, I. *Angew. Chem. Int. Ed.* **2008**, *47*, 3927.
